# The Value of H2BC12 for Predicting Poor Survival Outcomes in Patients With WHO Grade II and III Gliomas

**DOI:** 10.3389/fmolb.2022.816939

**Published:** 2022-04-25

**Authors:** Jie Zhou, Zhaoquan Xing, Yilei Xiao, Mengyou Li, Xin Li, Ding Wang, Zhaogang Dong

**Affiliations:** ^1^ Department of Nursing, Liaocheng Vocational and Technical College, Liaocheng, China; ^2^ Department of Urology, Qilu Hospital of Shandong University, Jinan, China; ^3^ Department of Neurosurgery, Liaocheng People’s Hospital, Liaocheng, China; ^4^ Department of Clinical Laboratory, Qilu Hospital of Shandong University, Jinan, China

**Keywords:** H2BC12, TCGA, diagnosis, prognosis, gliomas

## Abstract

**Purpose:** Glioma is a common primary malignant brain tumor. Grade II (GII) gliomas are prone to develop into anaplastic grade III (GIII) gliomas, which indicate a higher malignancy and poorer survival outcome. This study aimed to satisfy the increasing demand for novel sensitive biomarkers and potential therapeutic targets in the treatment of GII and GIII gliomas.

**Methods:** A TCGA dataset was used to investigate the expression of H2BC12 mRNA in GII and GIII gliomas and its relation to clinical pathologic characteristics. Glioma tissues were collected to verify results from the TCGA dataset, and H2BC12 mRNA was detected by RT-qPCR. ROC analysis was employed to evaluate the classification power for GII and GIII. The significance of H2BC12 mRNA GII and GIII gliomas was also investigated. In addition, H2BC12 expression-related pathways were enriched by gene set enrichment analysis (GSEA). DNA methylation level and mutation of H2BC12 were analyzed by the UALCAN and CBioPortal databases, respectively.

**Results:** Based on the sample data from multiple databases and RT-qPCR, higher expression of H2BC12 mRNA was found in GII and GIII glioma tissue compared to normal tissue, which was consistent with a trend with our clinical specimen. H2BC12 mRNA had a better power in distinguishing between GII and GIII and yielded an AUC of 0.706 with a sensitivity of 76.9% and specificity of 81.8%. Meanwhile, high H2BC12 levels were associated with IDH status, 1p/19q codeletion, primary therapy outcome, and the histological type of gliomas. Moreover, the overall survival (OS), disease-specific survival (DSS), and progress-free interval (PFI) of GII glioma patients with higher levels of H2BC12 were shorter than those of patients with lower levels as well as GIII patients. In the multivariate analysis, a high H2BC12 level was an independent predictor for poor survival outcomes of gliomas. The Wnt or PI3K-AKT signaling pathways, DNA repair, cellular senescence, and DNA double-strand break repair were differentially activated in phenotypes that were positively associated with H2BC12. H2BC12 DNA methylation was high in TP53 nonmutant patients, and no H2BC12 mutation was observed in gliomas patients.

**Conclusion:** H2BC12 is a promising biomarker for the diagnosis and prognosis of patients with WHO grade II and III gliomas.

## Introduction

Gliomas are tumors that occur at glial cells, which are important for cerebral nerve cells. They constitute the most prevalent primary brain cancer malignancy (Kiran et al., 2019). According to World Health Organization (WHO) classifications, histologically confirmed gliomas can be categorized into four grades: I, II, III, and IV. This is crucial for appropriate therapeutic strategies or clinical outcomes. Low-grade gliomas (LGG) show highly variable clinical behaviors (Cancer Genome Atlas Research Network et al., 2015) and correlate to a more favorable survival outcome. However, they still carry a 70% risk of disease progression within 10 years (Kiran et al., 2019). Aggressive and proliferative high-grade gliomas (HGG) show an unfavorable course, even when treated by surgical resection, radiotherapy, or chemotherapy that could prolong survival (Stupp et al., 2005; Huang et al., 2017). Treatment and prognosis also differ substantially among the four grades of glioma. It is worth noting that grade II (GII) gliomas are traditionally considered to have a low degree of malignancy, and they are prone to developing into anaplastic grade III (GIII) gliomas, indicating a higher malignancy with huge social, and medical burdens. Unfortunately, GIII exhibits invasive growth and complex pathological processes due to the lack of biomarkers for diagnosis and individualized treatment. GIII is associated with very poor survival outcomes in comparison to GII, and this has important therapeutic implications (Beppu et al., 2011; Suzuki et al., 2015). Thus, discriminating between GII and GIII gliomas is very important. However, the clinical reality is that clinicians often face difficulty when determining whether a patient has a GII or GIII glioma even if they have the patient’s histopathology results. Much scientific research combines GII and GIII as low-grade gliomas, while fewer studies have investigated the difference between GII and GIII, such as differences in survival outcome, key drivers of survival, and biomarkers, etc. The various clinical biomarkers currently used, such as O6-methylguanine-DNA methyltransferase (MGMT), have insufficient sensitivity, and specificity when it comes to gliomas (Wick et al., 2014). Several novel biomarkers for the diagnosis and prognosis of gliomas have been explored, including YPEL1 (Li et al., 2022) and ELK3 (Liu et al., 2021). However, these biomarkers are still not available for clinical use. Therefore, we must find novel biomarkers with high sensitivity and specificity urgently to improve the early diagnosis and molecular-targeted therapy of patients with gliomas.

It is known that a genetic predisposition for tumorigenesis is always accompanied by epigenetic alterations. Genome instability is characterized by the accumulation of genetic alterations such as point mutations, copy number alterations, or changes in chromosome numbers, and structures (Hanahan and Weinberg, 2011). For example, aberrant histone modifications can potentially enhance the oncogenic drivers in disease progression, metastatic potential, and resistance to therapy (Müller and Almouzni, 2017). Structurally, histone modification-related proteins are responsible for the compact chromatin in nucleosomes and can be modified via diverse enzymes, including histone family genes (H2A, H2B, H3, and H4), two heterodimers (H2A and H2B), and one DNA-associated H3/H4 tetramer (Sansó et al., 2012). Heterodimers H2A and H2B are important in chromatin-related processes including transcription, DNA replication, and repair (Moyal et al., 2011; Chen et al., 2012). It has been established that H2B monoubiquitination (H2Bub1) at lysine 120 is vitally significant in proper DNA repair, and lacking H2Bub1 is associated with abnormal H2AX phosphorylation, resulting in durable DNA damage response (Kari et al., 2011; Sadeghi et al., 2014). Notably, RNF20/40, ubiquitin ligases indispensable for H2Bub1 were also a part of tumorigenesis (Sethi et al., 2018; Zhou et al., 2021). Recent research indicated that low H2Bub1 expression was prognostic for disease progression, which supported the role of H2Bub1 as a tumor suppressor (Tarcic et al., 2017). Furthermore, loss of H2Bub1 was associated with poor differentiation, cancer stemness, and enhanced malignancy of non-small cell lung cancer (Zhang et al., 2019). Based on the above findings, histone genes might play a crucial role in tumorigenesis, and progression.

Here, H2B Clustered Histone 12 (H2BC12) was investigated in GII and GIII glioma tissue, assessing biomarkers for gliomas, associations with clinical characteristics, prediction survival outcome values, and the involved biological pathways. The methylation level and mutation of H2BC12 were also analyzed. Our findings suggested that H2BC12 might be recognized as a promising biomarker for the prognosis of GII and GIII gliomas.

## Materials and Methods

### Data Acquisition

Target RNA-seq data in TPM format, which were documented in TCGA and GTEx databases, were jointly processed by Toil workflow software (Vivian et al., 2017) and then downloaded from UCSC XENA (https://xenabrocwser.net/datapages/). TCGA database was searched for GII and GIII gliomas tissue (*n* = 528) and GTEx database was consulted to obtain matched normal tissue (*n* = 1,152). RNA-seq data were log2 transformed. Corresponding clinical data were also obtained. The inclusion criteria were defined as WHO GII or GIII classified patients with complete prognostic information.

### Inclusive and Exclusive Criteria of Enrolled Patients for the Construction of Risk Signature

The inclusive criteria of patients with gliomas for model construction were as follows: 1) patients with primary gliomas; 2) pathologic types of WHO II or III grade; 3) complete clinicopathological parameters; 4) only samples with RNA-sequencing data; 5) overall survival (OS) as the primary endpoint; 6) minimum follow-up of 90 days. The exclusion criteria included 1) patients with recurrent gliomas and 2) incomplete survival status and clinical information.

### GSEA Analysis

Hallmark gene set collections, including C2. cp.v7.2. symbols.gmt [Curated] and h. all.v7.2. symbols.gmt [Hallmarks], were retrieved from the Molecular Signatures Database (MSigDB) and chosen as target sets. Correlations between H2BC12 expression and all genes were characterized by R (v.3.6.3), followed by GSEA analysis using R package clusterProfiler (Yu et al., 2012). The significance threshold was set to |ES|>1, p. adjust<0.05, and FDR<0.25.

### Analysis of Immune Infiltration and Immune Regulatory Factor

From Bindea’s investigation (Bindea et al., 2013), the marker gene of 24 immune cells was retrieved. Based on mRNA TPM data, single-sample GSEA (ssGSEA) (Finotello and Trajanoski, 2018) was utilized to quantify the number of tumor-infiltrating immune cells. Spearman correlation was used to determine the relationship between H2BC12 and 24 cells. The ggplot2 package was used to create the figures. Moreover, the correlation between H2BC12 and immune regulatory factors, such as immune inhibitors, immune stimulators, and the MHC molecule from the TISIDB databases (http://cis.hku.hk/TISIDB/), was also analyzed.

### DNA Methylation Level and Mutation Analysis of H2BC12

The UALCAN database (Chandrashekar et al., 2017) (http://ualcan.path.uab.edu/index.html) was used to analyze the correlation between the DNA methylation level of the H2BC12 promoter region and the clinical characterization of gliomas. The CBioPortal database (Gao et al., 2013) (http://www.cbioportal.org/) was used to analyze H2BC12 mutation in patients with gliomas.

### RNA Extraction and Quantitative Real-Time RT-qPCR

Glioma tissues were collected from the Department of Neurosurgery, Liaocheng People’s Hospital (Shandong, China), and they included tissues from 22 GII and 26 GIII gliomas. Tissue RNAs were extracted using the RNAprep pure FFPE kit [cat. no. DP439, TIANGEN Biotech (Beijing) Co., Ltd.] according to instructions. The All-in-one™ First-Strand cDNA Synthesis kit (cat. no. QP006, GeneCopoeia, Inc.) was used to reverse-transcribe an equal amount of total RNA from each sample to cDNA. H2BC12 was detected using the CFX96 qPCR instrument (Bio-Rad Laboratories, Inc.) with the All-in-one™ qPCR Mix (cat. no. QP001, GeneCopoeia, Inc.). The primers for H2BC12 were as follows: forward 5′-AGA​AGG​GCT​CGA​AGA​AAG​CC-3′, reverse 5′-ATG​GTC​GAG​CGC​TTG​TTG​TA-3'. The size was 235 bp. The primers for GAPDH were as follows: forward 5′-GAA​GGT​GAA​GGT​CGG​AGT​C-3′, reverse 5′-GAA​GAT​GGT​GAT​GGG​ATT​TC-3'. The size was 225 bp. The conditions were as follows: following initial denaturation at 95°C 10 min, then 40 cycles of 95°C for 15 s, 62°C for 20 s, and 72°C for 10 s. The amplification specificity was determined by melting curve analysis. Data were normalized to GAPDH, and relative expression levels were evaluated using the 2^−ΔΔCT^ method.

### Statistical Analysis

R (v.3.6.3) was run to complete all statistical analyses. The diagnostic receiver operating characteristic (ROC) curve was generated using package pROC, while the time-dependent ROC (tROC) curve was plotted with assistance from package timeROC. Differential expression of H2BC12 in gliomas versus normal was statistically analyzed via Wilcoxon rank-sum tests. For correlational analysis between H2BC12 mRNA and clinicopathologic characteristics, tumor samples were assigned to two cohorts representative of high and low H2BC12 expression, respectively, with the cutoff value being the median H2BC12 expression of all samples. A Chi-square test was implemented to identify significance. Comparisons between two sets of data were completed by a Wilcoxon rank-sum test for two groups or the Kruskal–Wallis test when there were three groups or more. Prognostic significance of H2BC12 mRNA expression and clinicopathologic characteristics for overall survival (OS) of gliomas patients were identified by univariate and multivariate Cox regression analysis. The survival significance of H2BC12 mRNA expression in subgroups of clinicopathologic characteristics was investigated by stratification and Kaplan-Meier analysis. *p* value < 0.05 was considered statistically significant.

## Results

### Clinical Characteristics

The expression of H2BC12 mRNA and the corresponding clinicopathologic characteristics of 528 primary tumors were obtained from the glioma dataset; of these, 523 RNA-seq datasets were available. Matched clinical data were retrieved: WHO grade II and III, IDH status, 1p/19q codeletion, primary therapy outcome, gender, race, age, histological type, laterality, and OS event (Table 1).

**TABLE 1:** Characteristics of patients with gliomas based on TCGA.

Characteristic	Levels	Overall
n		528
WHO grade, n (%)	GII	224 (48%)
	GIII	243 (52%)
IDH status, n (%)	WT	97 (18.5%)
	Mut	428 (81.5%)
1p/19q codeletion, n (%)	codel	171 (32.4%)
	non-codel	357 (67.6%)
Primary therapy outcome, n (%)	PD	110 (24%)
	SD	146 (31.9%)
	PR	64 (14%)
	CR	138 (30.1%)
Gender, n (%)	Female	239 (45.3%)
	Male	289 (54.7%)
Race, n (%)	Asian	8 (1.5%)
	Black or African American	22 (4.3%)
	White	487 (94.2%)
Age, n (%)	≤40	264 (50%)
	>40	264 (50%)
Histological type, n (%)	Astrocytoma	195 (36.9%)
	Oligoastrocytoma	134 (25.4%)
	Oligodendroglioma	199 (37.7%)
Laterality, n (%)	Left	256 (48.9%)
	Midline	6 (1.1%)
	Right	261 (49.9%)
OS event, n (%)	Alive	392 (74.2%)
	Dead	136 (25.8%)
DSS event, n (%)	Alive	397 (76.3%)
	Dead	123 (23.7%)
PFI event, n (%)	Alive	318 (60.2%)
	Dead	210 (39.8%)

### High Expression of H2BC12 mRNA in Grade II and III Gliomas Tissue

**FIGURE 1:**
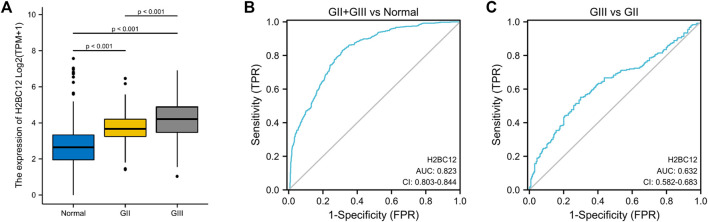
The expression of H2BC12 mRNA in normal, GII, and GIII glioma tissue and its clinical value as a biomarker for distinguishing between GII and GIII gliomas. H2BC12 showed significantly higher expression in GII or GIII tissue versus normal tissue **(A)**, p < 0.001. The diagnostic ROC curve showed the accurate discriminative capability of H2BC12 in distinguishing between normal and GII + GIII (AUC = 0.823). **(B)** ROC analysis of H2BC12 in classification power for GII and GIII (AUC = 0.632).

Apart from gliomas samples acquired, matched normal samples (*n* = 1,152) were obtained from the GTEx database. H2BC12 mRNA was examined in two cohorts, showing a significant upward trend in primary tumor tissue. Furthermore, the level of H2BC12 mRNA in GIII gliomas was higher than that of GII gliomas (Figure 1A, *p* < 0.001). And H2BC12 of GIII and GII were both higher than normal. Results of our clinical specimen showed a similar trend between GII and GIII (Supplementary Figure S1A, *p* < 0.05). These results revealed that H2BC12 might be an oncogene in gliomas.

### ROC Analysis for H2BC12 as a Biomarker of Grade II and III Gliomas

ROC curve was plotted to evaluate the diagnostic significance of H2BC12 mRNA for gliomas. The area under the curve (AUC) was 0.823 with 83.0% sensitivity and 68.4% specificity (Figure 1B), indicating significance in distinguishing between normal and tumor samples with certain accuracy. Furthermore, ROC analysis was also performed to compare GII and GIII gliomas. As shown in Figure 1C, AUC was 0.632, and the corresponding sensitivity and specificity were 56.5 and 72.5%, achieving a classification power for GIII and GII. The results of our clinical specimen also revealed that the AUC was 0.706 with a sensitivity of 76.9% and specificity of 81.8% (Supplementary Figure S1C). It seems that the results from our clinical specimen were better than from the dataset. This indicated that H2BC12 mRNA might be a more reliable biomarker.

### Correlations Between H2BC12 mRNA and Clinicopathologic Characteristics of Gliomas

**FIGURE 2:**
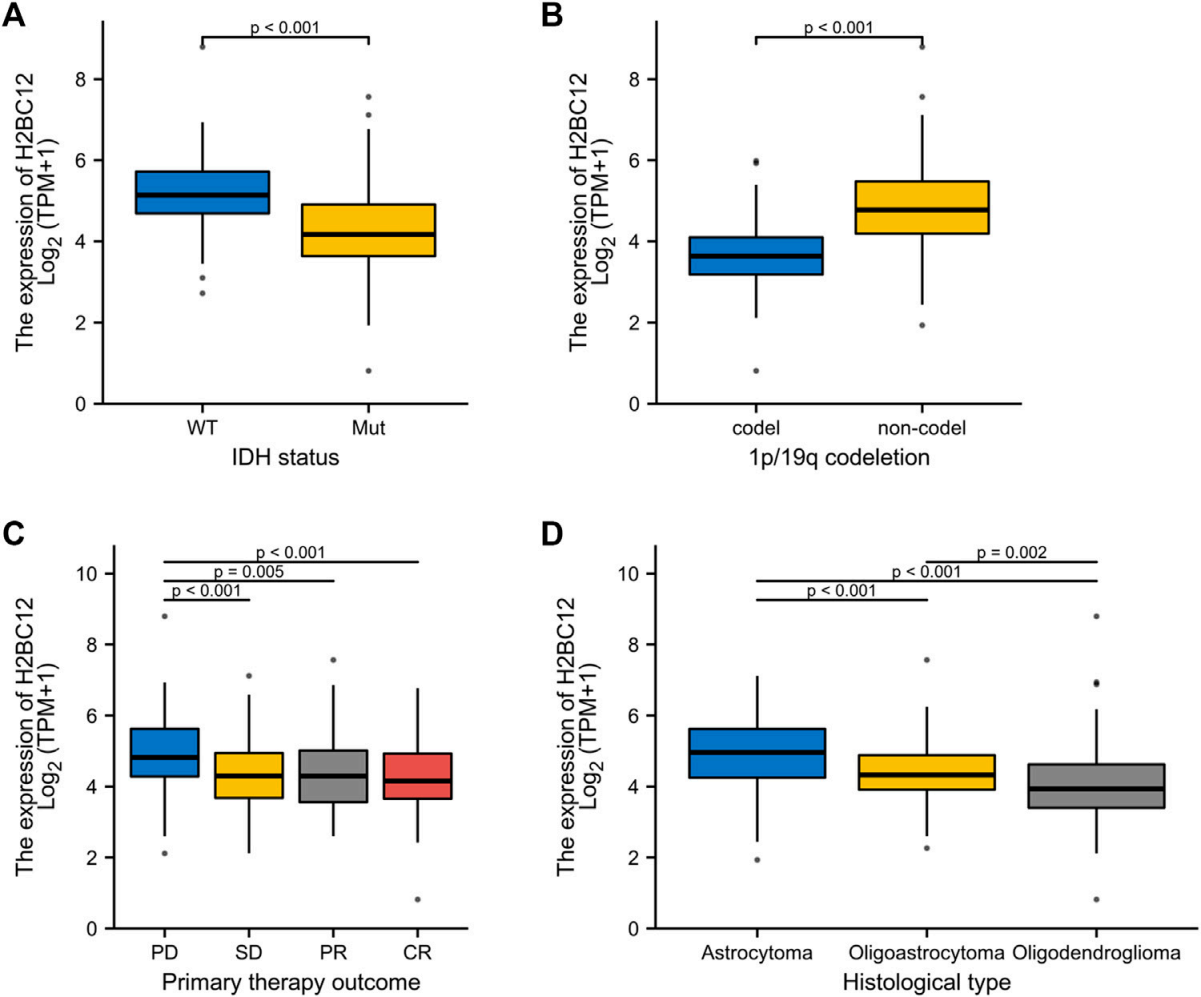
Association between H2BC12 expression and clinicopathologic characteristics. H2BC12 expression correlated significantly with IDH status **(A)**, p < 0.001, 1p/19q codeletion **(B)**, p < 0.001, primary therapy outcome **(C)**, p < 0.01, and histological type **(D)**, p < 0.01.

The correlational analysis demonstrated that there were significant associations between the H2BC12 mRNA and clinicopathologic characteristics, including IDH status, 1p/19q codeletion, primary therapy outcome, and histological type (Figures 2A–D). Our clinical results showed that H2BC12 mRNA was significantly correlated with IDH status, which was consistent with the conclusions drawn from the TCGA database (Supplementary Figure S1B, *p* < 0.05). In addition, tumor samples of each clinicopathologic subgroup were divided into two groups according to the median H2BC12 mRNA. Further analysis revealed that high H2BC12 mRNA expression was significantly associated with WHO grade, IDH status, 1p/19q codeletion, primary therapy outcome, histological type, OS event, disease-specific survival (DSS) event, and progress-free interval (PFI) event (Table 2, p < 0.001). Collectively, H2BC12 mRNA expression is intimately correlated with clinicopathologic features, suggesting that H2BC12 might be involved in glioma progression.

**TABLE 2:** Relationship between H2BC12 mRNA expression and clinical characteristics in gliomas.

Characteristic	Levels	Low expression of H2BC12	High expression of H2BC12	*p*
n		264	264	<0.001
WHO grade, n (%)	GII	138 (29.6%)	86 (18.4%)	
	GIII	95 (20.3%)	148 (31.7%)	
IDH status, n (%)	WT	13 (2.5%)	84 (16%)	<0.001
	Mut	250 (47.6%)	178 (33.9%)	
1p/19q codeletion, n (%)	codel	148 (28%)	23 (4.4%)	<0.001
	non-codel	116 (22%)	241 (45.6%)	
Primary therapy outcome, n (%)	PD	33 (7.2%)	77 (16.8%)	<0.001
	SD	76 (16.6%)	70 (15.3%)	
	PR	36 (7.9%)	28 (6.1%)	
	CR	84 (18.3%)	54 (11.8%)	
Gender, n (%)	Female	117 (22.2%)	122 (23.1%)	0.727
	Male	147 (27.8%)	142 (26.9%)	
Race, n (%)	Asian	4 (0.8%)	4 (0.8%)	0.230
	Black or African American	7 (1.4%)	15 (2.9%)	
	White	245 (47.4%)	242 (46.8%)	
Age, n (%)	≤40	131 (24.8%)	133 (25.2%)	0.931
	>40	133 (25.2%)	131 (24.8%)	
Histological type, n (%)	Astrocytoma	61 (11.6%)	134 (25.4%)	<0.001
	Oligoastrocytoma	69 (13.1%)	65 (12.3%)	
	Oligodendroglioma	134 (25.4%)	65 (12.3%)	
Laterality, n (%)	Left	122 (23.3%)	134 (25.6%)	0.412
	Midline	2 (0.4%)	4 (0.8%)	
	Right	137 (26.2%)	124 (23.7%)	
OS event, n (%)	Alive	237 (44.9%)	155 (29.4%)	<0.001
	Dead	27 (5.1%)	109 (20.6%)	
DSS event, n (%)	Alive	240 (46.2%)	157 (30.2%)	<0.001
	Dead	23 (4.4%)	100 (19.2%)	
PFI event, n (%)	Alive	196 (37.1%)	122 (23.1%)	<0.001
	Dead	68 (12.9%)	142 (26.9%)	

### Role of H2BC12 in Grade II and III Glioma Patient Survival

**FIGURE 3:**
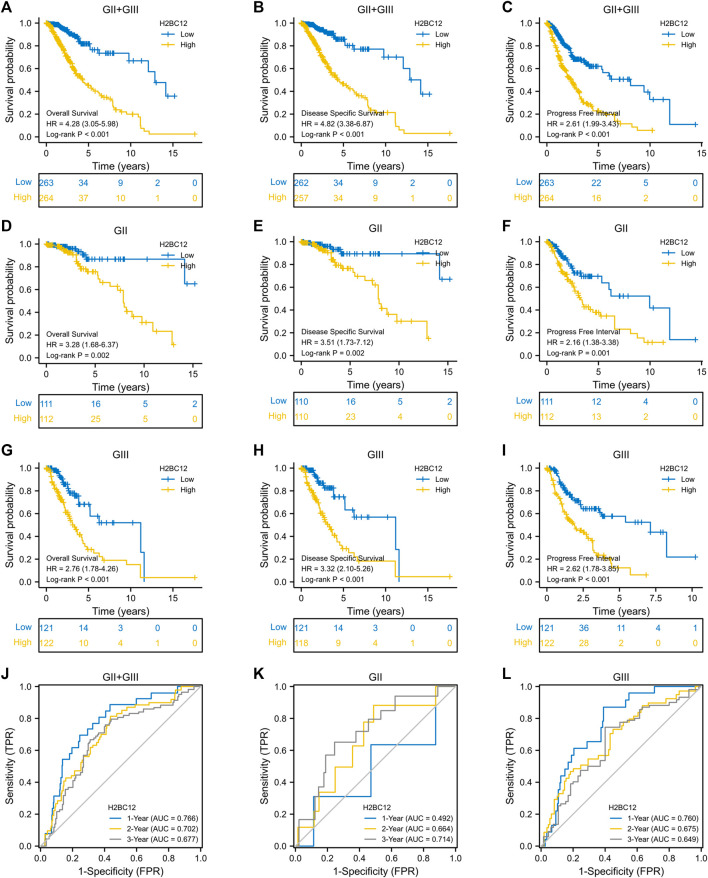
High expression of H2BC12 is associated with poor OS, DSS, and PFI in patients with GII and/or GIII. OS **(A)**, p < 0.001, DSS **(B)**, p < 0.001, and PFI **(C)**, p < 0.001 were significantly poorer in GII + GIII patients with high H2BC12 expression than those with low H2BC12 expression. Furthermore, OS, DSS, and PFI of GII **(D–F)** and GIII **(G–I)** were analyzed respectively. OS, Overall Survival; DSS, Disease-Specific Survival; PFI, Progress-Free Interval. **(J)** tROC curve demonstrated AUC values for 1-, 2-, and 3-years survival in GII + GIII as 0.766, 0.702, and 0.677, respectively. The 1-, 2-, and 3-years AOC values in GII were 0.492, 0.664, and 0.714 **(K).** The 1-, 2-, and 3-years AOC values in GIII were 0.760, 0.675, and 0.6499 **(L)**.

Gliomas were considered to have different degrees of malignancy and survival outcomes. However, few studies investigated the relationship between gene expression and survival outcomes for GII and GIII separately. First, we explored the role of H2BC12 in survival outcomes, and Figure 3A shows that the OS of GII + GIII patients with high H2BC12 expression was much poorer compared to those with low H2BC12 expression (p < 0.001). Similar results were also observed as regards DSS and PFI (Figures 3B,C, p < 0.001). The prognostic value of H2BC12 in GII or GIII was further evaluated. Figures 3D–F shows that OS, DSS, and PFI of GII gliomas with higher levels of H2BC12 were shorter than those with lower levels [HR = 3.28 (1.68–6.37) for OS, HR = 3.51 (1.73–7.12) for DSS, and HR = 2.16 (1.38–3.38) for PFI]. A similar trend was also shown in GIII patients, and HR was 2.76 (1.78–4.26) for OS, 3.32 (2.10–5.26) for DSS, and 2.62 (1.78–3.85) for PFI (Figures 3G–I, *p* < 0.001). Besides, the tROC curves were drawn to identify the predictive ability of H2BC12 mRNA for OS of GII and/or GIII patients. The AUC values for 1-, 2-, and 3-years OS of GII + GIII were 0.766, 0.702, and 0.677, respectively (Figure 3J). The AUC values for 1-, 2- and 3-years GII were 0.492, 0.664, and 0.714 (Figure 3K). The AUC values for 1-, 2-, and 3-years GIII were 0.760, 0.675, and 0.6499 (Figure 3L). To identify the prognostic factors for OS of gliomas patients, univariate regression analysis was performed using a Cox model, demonstrating significant prognostic significance of H2BC12 mRNA, WHO grade, 1p/19q codeletion, TP53, IDH status, age, and histological type for OS (Table 3, p < 0.01). Additionally, a further multivariate model was established and revealed that H2BC12 mRNA, WHO grade, IDH status, age, and histological type had independent prognostic significance for gliomas OS (Table 3, p < 0.05). It was suggested that H2BC12 was equipped with a good prognostic performance.

**TABLE 3:** Correlations between overall survival and mRNA expression of H2BC12 analyzed by univariate and multivariate Cox regression.

Characteristics	Total(N)	Univariate analysis		Multivariate analysis	
		Hazard ratio (95% CI)	*p* value	Hazard ratio (95% CI)	*p* value
WHO grade (GIII vs. GII)	466	3.059 (2.046–4.573)	**<0.001**	1.845 (1.147–2.967)	**0.012**
1p/19q codeletion (non-codel vs. codel)	527	2.493 (1.590–3.910)	**<0.001**	1.293 (0.670–2.496)	0.443
TP53 (High vs. Low)	527	1.689 (1.189–2.400)	**0.003**	1.352 (0.874–2.091)	0.175
IDH status (Mut vs. WT)	524	0.186 (0.130–0.265)	**<0.001**	0.455 (0.281–0.735)	**0.001**
Gender (Male vs. Female)	527	1.124 (0.800–1.580)	0.499		
Age (>40 vs. ≤40)	527	2.889 (2.009–4.155)	**<0.001**	3.491 (2.191–5.561)	**<0.001**
Histological type (Oligoastrocytoma&Oligodendroglioma vs. Astrocytoma)	527	0.606 (0.430–0.853)	**0.004**	1.018 (0.642–1.615)	0.939
H2BC12 (High vs. Low)	527	4.415 (2.885–6.756)	**<0.001**	2.267 (1.252–4.104)	**0.007**

Bold values indicates that the significant values (p ≤ 0.05).

### Clinical Stratification

**FIGURE 4:**
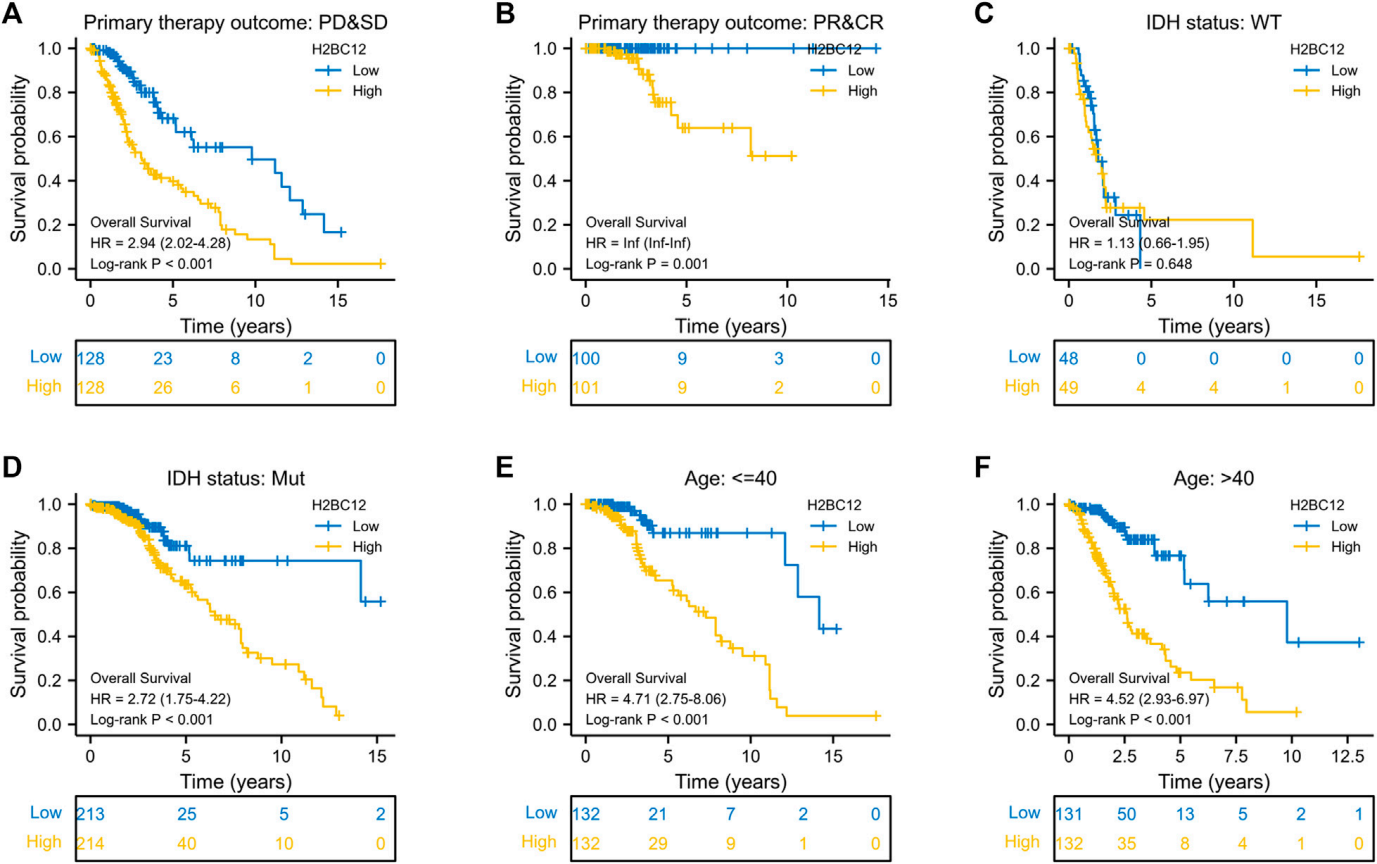
Clinical stratification analysis of the survival difference in the high- and low-H2BC12 groups by primary therapy outcome, IDH status, and age. Kaplan-Meier survival curves of patients in the high- and low-H2BC12 groups within eight clinically stratified subgroups, including primary therapy outcome: PD&SD **(A)**, primary therapy outcome: PR&CR **(B)**, IDH status: WT **(C)**, IDH status: Mut **(D)**, age<=40 **(E)** and age>40 **(F)**, respectively. Patients in the low-H2BC12 group had better survival outcomes than those in the high-H2BC12 group across all clinically stratified subgroups except the IDH status of WT (p < 0.01).

As proven in multivariate Cox regression analysis, primary therapy outcome, IDH status, age, and histological type were independent prognostic factors for glioma OS. Then, clinical stratification was conducted based on the glioma dataset; in subgroups of primary therapy outcomes PD&SD, primary therapy outcome PR&CR, IDH status: Mut, age < = 40, and age >40, patients with low H2BC12 expression had better survival outcomes than those with highly expressing H2BC12 (Figures 4A–F, p < 0.001). This reflected that H2BC12 had independent prognostic significance for glioma OS, and increased H2BC12 was associated with poorer OS.

### H2BC12-Related Signaling Pathways Based on GSEA

**FIGURE 5:**
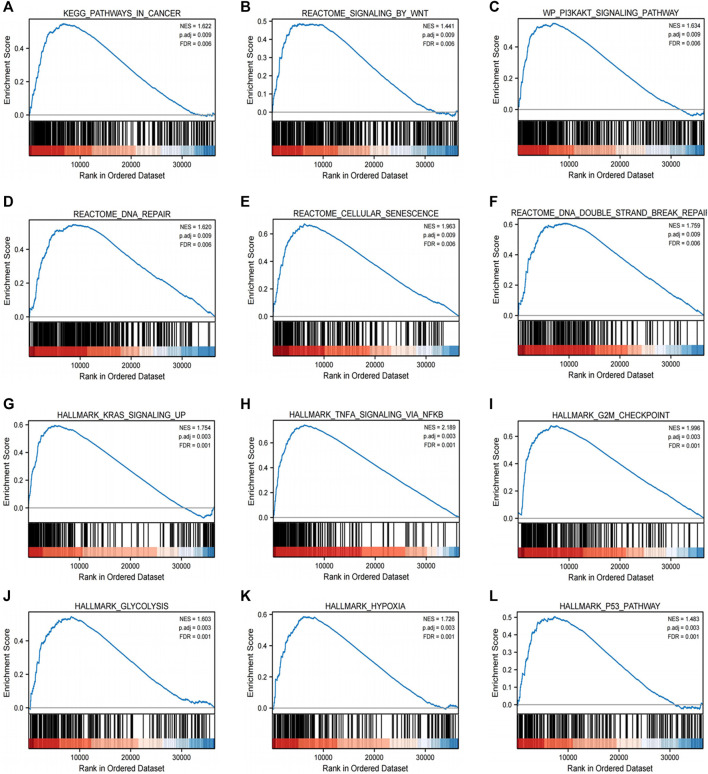
Enrichment plots from GSEA. GSEA results showing pathways in cancer **(A)**, signaling by wnt **(B)**, the PI3K-AKT signaling pathway **(C)**, DNA repair **(D)**, cellular senescence **(E)**, DNA double-strand break repair **(F)**, KRAS signaling up **(G)**, TNFA signaling via NFKB **(H)**, G2M checkpoint **(I)**, glycolysis **(J)**, hypoxia **(K)**, and the p53 pathway **(L)**, which are differentially enriched in H2BC12-high expression phenotype. NES, normalized ES; p. adj, p. adjust; FDR, False Discovery Rate.

GSEA was performed to find the activated signaling pathways related to H2BC12 in gliomas. Based on the curated collection, there were six signaling pathways activated in H2BC12 overexpressed phenotype, including pathways in cancer, Wnt or the PI3K-AKT signaling pathway, DNA repair, cellular senescence, and DNA double-strand break repair. Based on the Hallmarks collection defined by MSigDB, other than the above six pathways, the KRAS signaling up, TNFA signaling via NFKB, G2M checkpoint, glycolysis, hypoxia, and p53 pathways also presented with significant enrichment in H2BC12 overexpressed phenotype (Figure 5; Table 4). Collectively, H2BC12 mRNA might serve as an important player in the initiation and development of gliomas.

**TABLE 4:** Gene sets enriched in positively correlated with H2BC12 mRNA expression phenotype high.

MSigDB collection	Gene set name	NES	p.adj	FDR
c2.cp.v7.2.symbols.gmt [Curated]	KEGG_PATHWAYS_IN_CANCER	1.622	0.009	0.006
	REACTOME_SIGNALING_BY_WNT	1.441	0.009	0.006
	WP_PI3KAKT_SIGNALING_PATHWAY	1.634	0.009	0.006
	REACTOME_DNA_REPAIR	1.620	0.009	0.006
	REACTOME_CELLULAR_SENESCENCE	1.963	0.009	0.006
	REACTOME_DNA_DOUBLE_STRAND_BREAK_REPAIR	1.759	0.009	0.006
h.all.v7.2.symbols.gmt [Hallmarks]	HALLMARK_KRAS_SIGNALING_UP	1.754	0.003	0.001
	HALLMARK_TNFA_SIGNALING_VIA_NFKB	2.189	0.003	0.001
	HALLMARK_G2M_CHECKPOINT	1.996	0.003	0.001
	HALLMARK_GLYCOLYSIS	1.603	0.003	0.001
	HALLMARK_HYPOXIA	1.726	0.003	0.001
	HALLMARK_P53_PATHWAY	1.483	0.003	0.001

NES, normalized enrichment score; p.adj, adjust *p* value; FDR, false discovery rate.

### H2BC12 Expression Was Linked to the Level of Immune Infiltration and Immune Regulatory Factor

**FIGURE 6:**
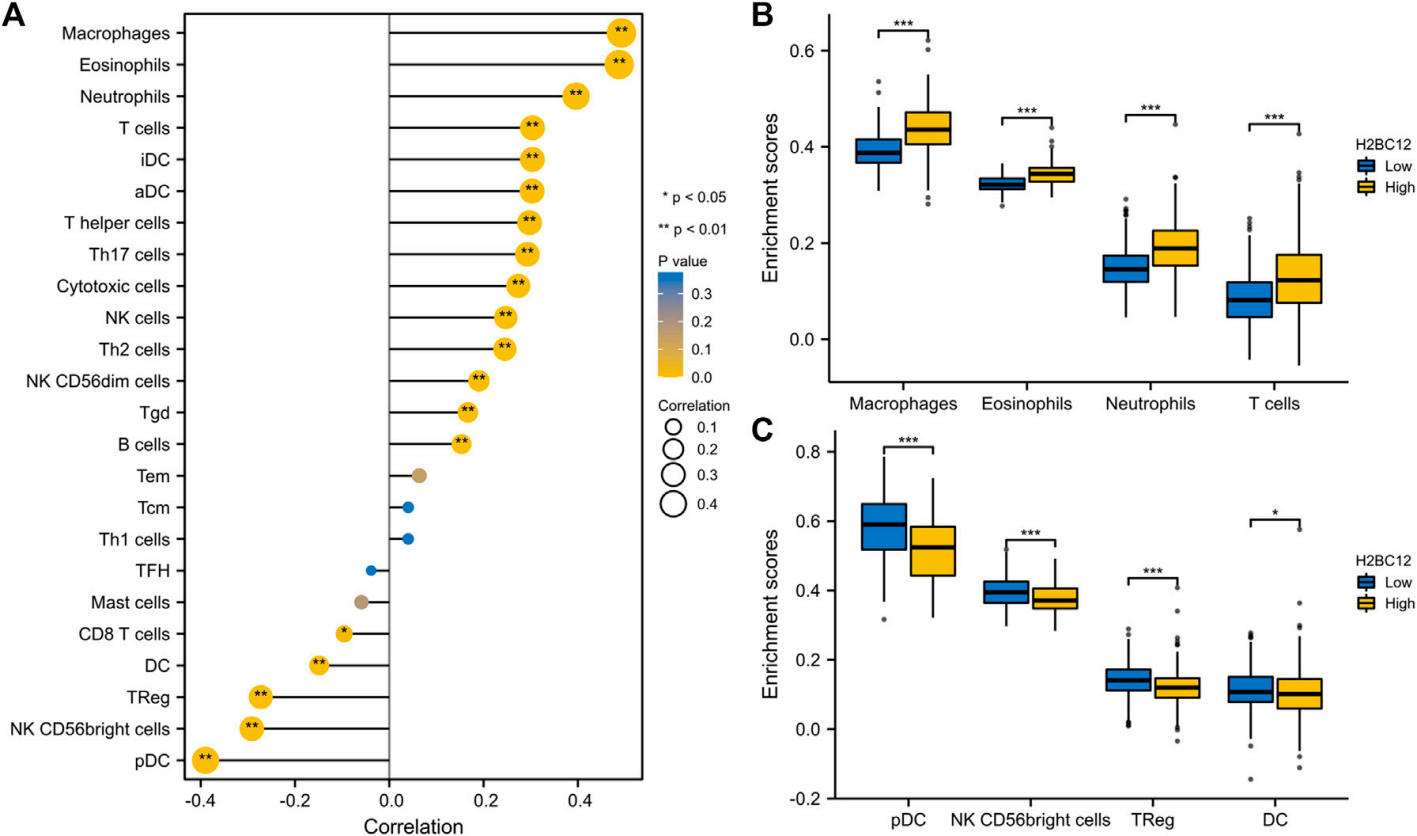
Correlation analysis between H2BC12 and immune infiltration. **(A)** Association analysis between H2BC12 expression and immune cells. **(B, C)** Differences in immune cell infiltration levels between high and low H2BC12 expression groups.

**FIGURE 7:**
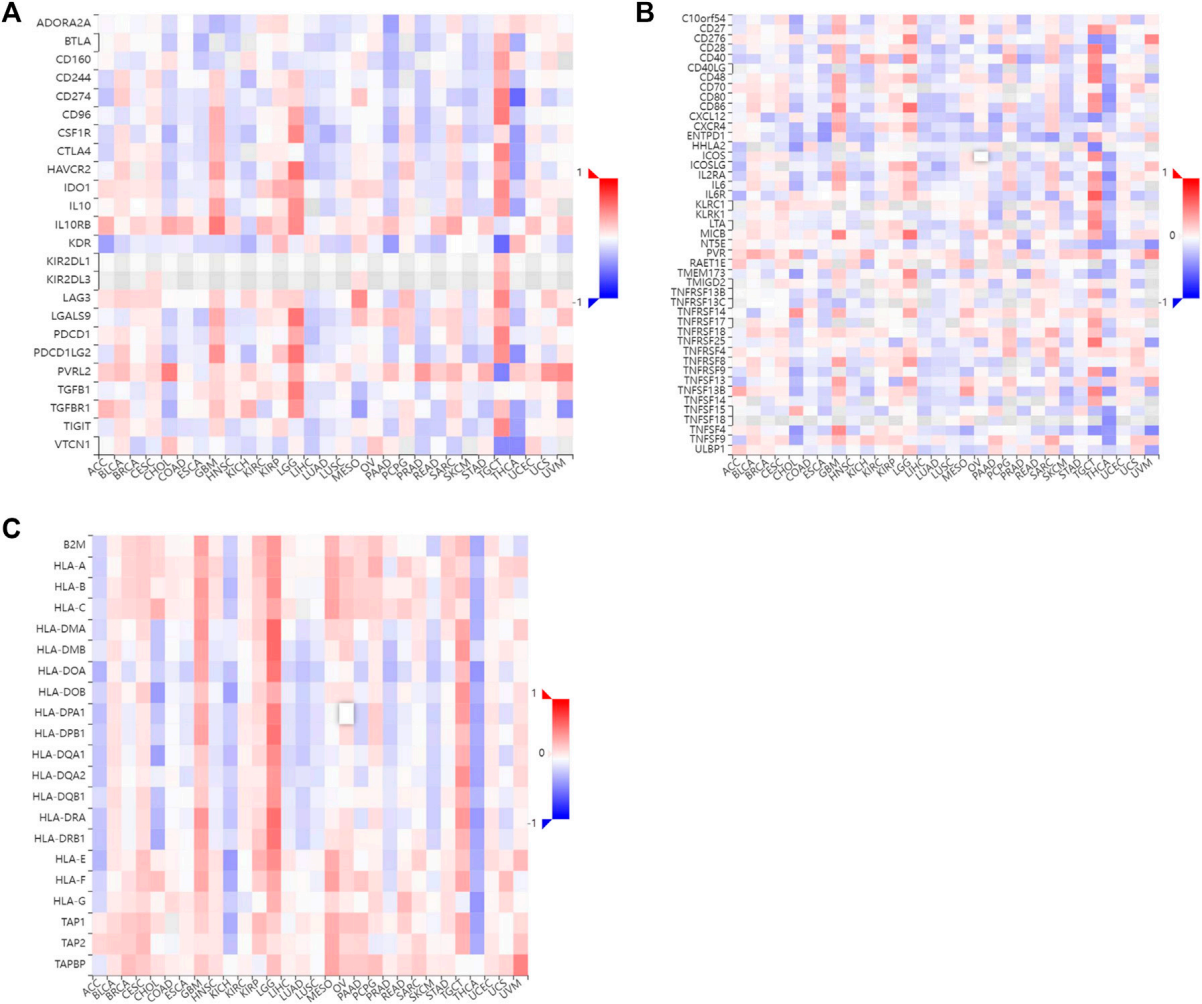
The relationship between H2BC12 and immune regulatory factor. The level of H2BC12 mRNA was positively correlated with immune inhibitors **(A)**, immune stimulators **(B),** and MHC molecules **(C)**. Red indicated a significant positive correlation, and blue indicated a significant negative correlation.

Tumor-infiltrating lymphocytes are independent indicators of cancer survival. As result, we evaluated whether H2BC12 was related to immune infiltrate in gliomas. According to our findings, H2BC12 showed a strong positive correlation with macrophages, eosinophils, neutrophils, and T cells; H2BC12 exhibited a strong inverse relationship with pDC, NK CD56bright cells, TReg, and DC (Figure 6A). Further analysis showed that compared with the low-H2BC12 group, the infiltration of Neutrophils and T cells in the high-H2BC12 group was significantly increased (Figure 6B). The infiltration levels of pDC, NK CD56bright cells, Treg, and DC were significantly reduced in the high-H2BC12 group (Figure 6C). Moreover, Results of the relationship of H2BC12 with immune regulatory factors showed that H2BC12 was positively correlated with immun inhibitors, including PDCD1LG2, LGALS9, and L10RB (Figure 7A), as well as immune stimulators, including CD40, CD86, and MICB (Figure 7B), and MHC molecules, including HLA-DMA, HLA-DMB, and HLA-DOA (Figure 7C).

### H2BC12 Promoter Methylation Level and Mutation Analysis

The level of DNA methylation in the H2BC12 promoter region in patients with TP53 nonmutant was significantly higher than that in patients with TP53 mutant (Supplementary Figure S2A). Moreover, the levels in those aged between 41 and 60 years were significantly higher than in those aged between 21 and 40 years (Supplementary Figure S2B). There have not been any significant differences in terms of gender or race yet (Supplementary Figure S2C, D). In addition, the H2BC12 mutation was not investigated in glioma patients and was very low in most brain tumors (Supplementary Figure S2E).

## Discussion

Gliomas are fatal tumors most prevalent in the central nervous system (CNS), and they are among the most devastating forms of cancer. Low-grade tumors grow slowly with lesser malignant properties than high-grade tumors (Perez and Huse, 2021; Zhao et al., 2021). However, there is a high risk of disease progression to advanced gliomas in most low-grade glioma patients (Kiran et al., 2019). It is well known that GII gliomas can easily develop into GIII gliomas, which leads to a poor survival outcome after receiving chemotherapy (Xiao et al., 2020). There are no suitable biomarkers to discriminate between GII and GIII gliomas. The role of survival outcome, key drivers of survival, etc. remains to be further explored. According to bioinformatics, the WHO included several molecular markers, such as IDH mutation status and chromosome 1p or 19q codeletion (1p/19q codeletion) status, into the guidelines for the diagnosis of gliomas to increase the accuracy in disease diagnosis and further treatment (Louis et al., 2016). In this context, the demand for biomarkers with prognostic and diagnostic values is increasing, which will be of vital significance for the treatment and prognosis of patients with GII and GIII gliomas.

In this study, we firstly obtained RNA-seq data documented in TCGA and matched normal samples from GTEx in the UCSC XENA database, demonstrating that H2BC12 mRNA significantly increased in tumor tissue compared to normal control. A similar trend was observed between GII and GIII and was also confirmed by the clinical specimen. These suggested that H2BC12 might be active in promoting glioma initiation. H2BC12 encoded a replication-dependent histone that was a member of the histone H2B family. H2B played a crucial role in chromatin-related processes involved in transcription, DNA replication, and repair. Kim et al. (Kim et al., 2012) reported the top six most highly expressed genes in breast cancer, including STAT3, CTSD, SREBF1, IGFBP5, and DDR1, from 49 signature genes of tumor dormancy based on cancer cell line data and microarray data, which further verified the role of H2BC12 as a potential tumor dormancy marker (Kim et al., 2012). Dormant cells are highly adaptable in chemotherapy since they can rapidly target proliferating cells. Meanwhile, they can still survive for a long time and even reproduce after chemotherapy is terminated. Han et al. (Han et al., 2019) reported that H2BC12 displayed increased expression in drug-resistant cell MDA-MB-231 in breast cancer, showing a close relationship between the H2BC12 and drug resistance. Here, we found that H2BC12 mRNA presented with high expression in gliomas compared with normal tissues, and its expression in GIII was also higher than in GII. This implied that H2BC12 might be a therapeutic target or biomarker and that it is involved in promoting glioma progression.

Research revealed that H2A and H2B are important participants in chromatin transcription, DNA replication, and repair (Li et al., 2017). Similarly, we noted the good diagnostic performance of H2BC12 for GII and GIII, characterized by an AUC of 0.823. Meanwhile, H2BC12 could distinguish GIII gliomas from GII gliomas with 76.9% sensitivity and 81.8% specificity, which might improve the diagnosis and therapy of gliomas. We then profiled the association between H2BC12 and clinicopathologic characteristics of gliomas. Notably, the increased H2BC12 was correlated significantly with IDH status, 1p/19q codeletion, primary therapy outcome, and histological type. This demonstrated that H2BC12 mRNA is closely related to the clinicopathologic characteristics of gliomas, and H2BC12 might be involved in disease progression.

The tROC curve also validated the moderate prognostic value of H2BC12 for OS of GII and/or GIII in 1, 2, and 3 years. This indicated that H2BC12 might predict the survival outcome of gliomas, which was consistent with a previous study that showed that signatures based on histone gene family are potentially good indicators for the outcome of cervical cancer patients (Li et al., 2017). It was worth noting that the AUC was different between GII and GIII. In GII, the AUC of 3 years was more than that of 2 years and then 1 year. However, the opposite trend was observed in GIII, and the AUC of 1 year was better than those of 2 or 3 years. This gave us a hint that H2BC12 had a different value for predicting survival outcomes in patients with GII and GIII. However, its predictive power was different for different years in GII and GIII, indicating H2BC12 might play an important role in gliomas progression. No previous studies have reported a link between H2BC12 and gliomas. Further survival analysis was conducted to validate the association of high H2BC12 expression with adverse survival outcomes of GII and GIII patients. Interestingly, the higher H2BC12, the shorter OS, DSS, and PFI of GII patients. A similar trend was also observed in GIII patients. We thus believe that H2BC12 serves as a high-risk factor for GII and GIII. Previous bioinformatics analysis identified that high H2BC12 predicted adverse outcomes of breast, pancreatic, and ovarian cancers (Li et al., 2018; Li and Zhan, 2019; Yu et al., 2020). We next performed univariate and multivariate analyses to identify factors predicting OS with Cox regression models. Results showed that H2BC12, WHO grade, IDH status, age, and histological type could all be prognostic factors for gliomas. Given this, a further clinical stratification analysis was designed to identify whether H2BC12 was an independent predictor.

Finally, we conducted GSEA to uncover the H2BC12-related pathways in gliomas. Results showed that there were six pathways, including pathways in cancer, the Wnt or PI3K-AKT signaling pathway, DNA repair, cellular senescence, and DNA double-strand break repair, which demonstrated differential enrichment in higher H2BC12. Research reveals that activated PI3K-AKT could facilitate the invasiveness of glioma cells (Li et al., 2019). DNA repair genes are associated with gliomas (Tang et al., 2018). DNA repair damage is the main cause of radio-resistance and chemo-resistance in gliomas (Zeng et al., 2019). A study suspected that targeting an H2Bub1 that regulates both transcription and DNA damage repair may inhibit an oncogenic transcriptional expression profile while simultaneously impairing the ability of the cell to effectively repair DNA damage, thereby increasing its sensitivity to a second drug that induces DNA damage (Jeusset and McManus, 2021). It has also been found that RNF20 (and RNF40) expression is increased in luminal B tumors, and er-positive tumors with high H2Bub1 abundance have poorer survival (Tarcic et al., 2017). All these findings indicate the potential important role of H2BC12 in gliomas progression. Moreover, as a new therapeutic strategy, immunotherapy, has drawn the attention of the field of gliomas. However, only a minority of glioma patients got responses due to a lacking of effective biomarkers (Chiocca et al., 2019). The current results showed that H2BC12 had a positive correlation to immune cells, including macrophages, NK cells, Treg, and T cells. These findings gave us a hint that H2BC12 might be involved in the immunoregulation of gliomas, which was consistent with a previous study that DNAJC10 was correlated with immune cell infiltrations and immune checkpoint genes (Liu et al., 2022) as well as the replication factor C2 (Zhao et al., 2022). Furthermore, our results also showed that H2BC12 was positively associated with immune regulatory factors, including immune inhibitor PDCD1LG2, immune stimulator CD40, and MHC molecule HLA-DMA. H2BC12 could be a potential prognostic marker and immunotherapy marker in gliomas.

In all, this study verified the significance of H2BC12 in the diagnosis and prognosis of GII and GIII gliomas. Inevitably, limitations still exist. First, the study was carried out only with bioinformatics analysis, requiring further validation in clinical samples. Second, there is a need to clarify the H2BC12-mechanism of action.

## Conclusion

This study identified the differentially up-regulated expression of H2BC12 in GII and GIII glioma tissue and proved its significant ability in predicting the adverse overall survival of GII and GIII gliomas patients. H2BC12, therefore, has promising application for the diagnosis and prognosis of gliomas.

## Data Availability

The datasets presented in this study can be found in online repositories. The names of the repository/repositories and accession number(s) can be found in the article/Supplementary Material.

## Supplementary Material

Supplementary Figure S1 The expression and clinical value of H2BC12 mRNA in GII and GIII gliomas tissues. H2BC12 showed significantly higher expression in GIII tissue than in GII tissue **(A)**, p<0.05 and was related to IDH status **(B)**, p<0.05. **(C)** ROC analysis of H2BC12 in classification power for GII and GIII (AUC=0.706).

Supplementary Figure S2 H2BC12 promoter methylation level and mutation analysis. **(A–D)** The level of DNA methylation in the H2BC12 promoter region in patients with TP53 mutant status, age, gender, and race. **(E)** H2BC12 mutation in most brain tumors. ns: P>0.05; *P<0.05.

## Abbreviations

WHO, World Health Organization; GSEA, gene set enrichment analysis; ssGSEA, single sample gene set enrichment analysis; H2Bub1, H2B monoubiquitination; H2BC12, H2B Clustered Histone 12; MSigDB, Molecular Signatures Database; ssGSEA, single-sample GSEA; ROC, receiver operating characteristic; tROC, time-dependent ROC; OS, overall survival; AUC, area under the curve; DSS, disease-specific survival; PFI, progress-free interval; FDR, False Discovery Rate; WT, wild type; MUT, mutant type.
