# Supplementary figures and images for: The Value of H2BC12 for Predicting Poor Survival Outcomes in Patients With WHO Grade II and III Gliomas
Source: Front Mol Biosci. 2022 Apr 25;9:816939. doi: 10.3389/fmolb.2022.816939 (PMC9081347; doi:10.3389/fmolb.2022.816939)

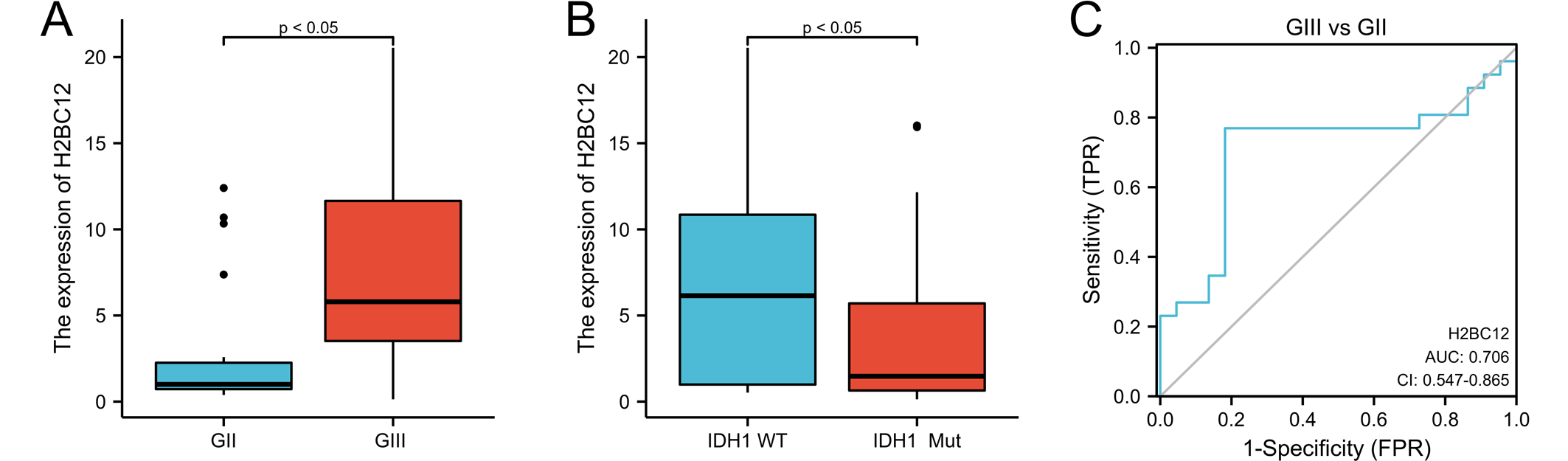

Supplement: Supplementary file 1 [file Image1.tiff]

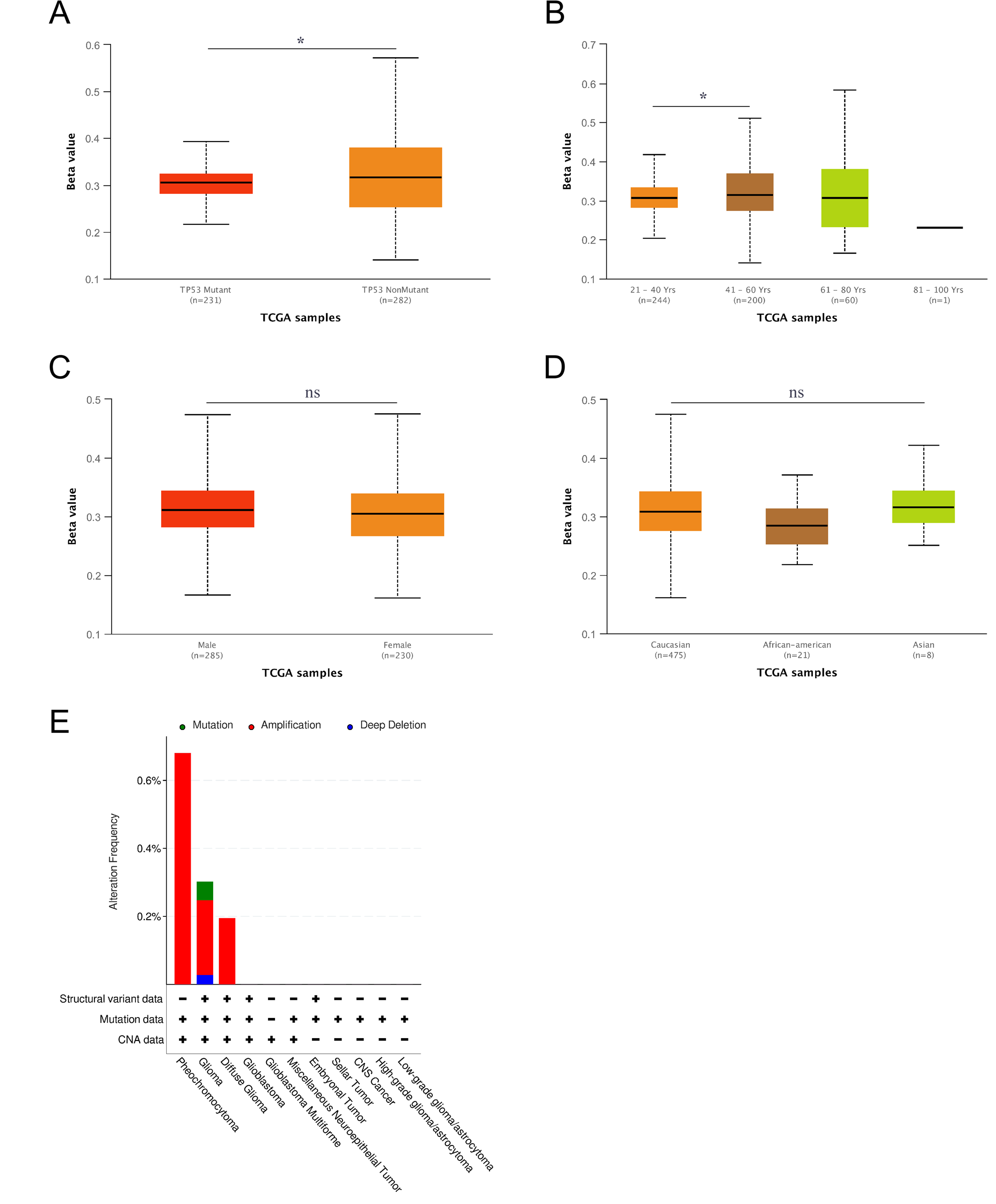

Supplement: Supplementary file 2 [file Image2.tif]
